# Estimands for clinical endpoints in tuberculosis treatment randomized controlled trials: a retrospective application in a completed trial

**DOI:** 10.1186/s13063-024-07999-w

**Published:** 2024-03-12

**Authors:** Isabelle R. Weir, Suzanne M. Dufault, Patrick P. J. Phillips

**Affiliations:** 1 Center for Biostatistics in AIDS Research in the Department of Biostatistics, Harvard T.H. Chan School of Public Health, Boston, MA USA; 2 UCSF Center for Tuberculosis, University of California, San Francisco, San Francisco, CA USA; 3 Division of Biostatistics, Department of Epidemiology and Biostatistics, University of California, San Francisco, San Francisco, CA USA

**Keywords:** Clinical trials, Estimands, Tuberculosis

## Abstract

**Background:**

Randomized trials for the treatment of tuberculosis (TB) rely on a composite primary outcome to capture unfavorable treatment responses. However, variability between trials in the outcome definition and estimation methods complicates across-trial comparisons and hinders the advancement of treatment guidelines. The International Council for Harmonization (ICH) provides international regulatory standards for clinical trials. The estimand framework outlined in the recent ICH E9(R1) addendum offers a timely opportunity for randomized trials of TB treatment to adopt broadly standardized outcome definitions and analytic approaches. We previously proposed and defined four estimands for use in this context. Our objective was to evaluate how the use of these estimands and choice of estimation method impacts results and interpretation of a large phase III TB trial.

**Methods:**

We reanalyzed participant-level data from the REMoxTB trial. We applied four estimands and various methods of estimation to assess non-inferiority of both novel 4-month treatment regimens against standard of care.

**Results:**

With each of the four estimands, we reached the same conclusion as the original trial analysis that the novel regimens were not non-inferior to standard of care. Each estimand and method of estimation gave similar estimates of the treatment effect with fluctuations in variance and differences driven by the methods applied for handling intercurrent events.

**Conclusions:**

Our application of estimands defined by the ICH E9 (R1) addendum offers a formalized framework for addressing the primary TB treatment trial objective and can promote uniformity in future trials by limiting heterogeneity in trial outcome definitions. We demonstrated the utility of our proposal using data from the REMoxTB randomized trial. We outlined methods for estimating each estimand and found consistent conclusions across estimands. We recommend future late-phase TB treatment trials to implement some or all of our estimands to promote rigorous outcome definitions and reduce variability between trials.

**Trial registration:**

ClinicalTrials.gov NCT00864383. Registered on March 2009

**Supplementary Information:**

The online version contains supplementary material available at 10.1186/s13063-024-07999-w.

## Background

Tuberculosis (TB) remains a leading cause of death worldwide [1]. The 6-month standard of care treatment (a combination of isoniazid, rifampin, pyrazinamide, and ethambutol) is long and burdensome for persons with TB infection [2]. A current research focus is therefore to identify shorter novel treatment regimens that are no less efficacious than the current standard of care. Late-phase randomized controlled trials aim to assess a novel regimen against standard of care for the primary objective of comparing proportions of participants with long-term unfavorable outcomes. These trials continue to rely on a composite binary outcome measure [3]. Participants are typically followed at least a year after randomization for determination of a long-term clinically favorable or unfavorable outcome, the latter determined by the presence of events such as death, treatment failure, relapse, and recurrence. A recent systematic review of 31 TB treatment trials largely found consensus in the components of the composite outcome but heterogeneity in specific definitions [4]. There are also differences in the application of statistical methods used to carry out the primary analysis.

The International Council for Harmonization (ICH) guidelines provide established international regulatory standards for clinical trials. The estimand framework outlined in the recent ICH E9(R1) efficacy guideline addendum offers a timely opportunity for randomized trials of TB treatment to adopt a broadly standardized definition and analytic approach for this primary objective [5]. Harmonization through estimand specification will allow for easier and more insightful between-trial comparisons and formal meta-analysis. We offer a specification of how the estimand framework can be applied to TB treatment trials by defining four estimands to leverage a single trial to address the needs of different stakeholders. Our proposal includes a comprehensive set of intercurrent and missing data events reasonable to expect in this setting with estimand definitions that are already published [6]. The four estimands share this common set of potential events but differ by the selection and application of strategies for handling such events.

In this paper, we aim to demonstrate the utility of our proposed estimands with appropriate estimation methods for the primary efficacy objective in TB treatment trials by reanalyzing individual participant-level data from a large phase III trial. In the first section, we briefly review the ICH E9(R1) estimand framework and the four estimands from our proposal. We then discuss statistical estimation methods for handling intercurrent and missing data events (hereafter referred to collectively as ICEs), including specification of underlying assumptions and limitations. In the third section, we re-analyze the primary outcome data from the REMoxTB trial according to each estimand and applying different statistical methods of estimation [7]. We conclude with a discussion about the application of the estimand framework for TB treatment trial objectives and limitations of our proposal and illustration.

## Methods

### Estimands

The US FDA, among other regulatory agencies, adopted the ICH E9(R1) addendum on estimands and sensitivity analysis in clinical trials in May 2021. This addendum presents a structured framework to help define precise treatment effects in clinical trials. The work of constructing an estimand should occur during the protocol and design stage of the trial and should engage a diverse range of protocol team members representing different disciplines to ensure the proposed estimand(s) address the needs of different trial stakeholders. One important aim is to encourage explicit pre-specification of how the treatment effect will be captured including the statistical analysis methods and plans for handling inevitable imperfections in the data [5].

An estimand is explicitly defined by five attributes: (1) the treatment being tested and the alternative treatment to which it will be compared; (2) the population of patients targeted by the clinical question for whom the specified treatment is intended; (3) the endpoint, or variable, that will be obtained for each trial participant and will be used to determine the success or failure of the treatment; (4) the specification of intercurrent events that are likely to arise and how they will be handled in the analysis of the study; and (5) the population-level summary measure that will, through the pre-specified analysis, allow for a comparison of different treatment conditions.

For the primary efficacy objective of sustained clinical efficacy in TB treatment trials, we would define these attributes as follows. (1) The treatment attribute will be trial-specific and align with the experimental and control/standard of care regimens offered to participants through the trial. (2) The population will also depend on the target population of the specific trial and may be shaped by the inclusion and exclusion criteria. An example target population could be individuals aged more than 18 years with drug-susceptible pulmonary TB. (3) The participant-level endpoint is the determination of favorable or unfavorable long-term clinical efficacy. Our systematic review found general agreement between trials in the components for how to define unfavorable outcomes but differences in how the components were handled [4].

(4) The fourth attribute, specification of intercurrent events, demands the most forethought and attention. Intercurrent events are “events occurring after treatment initiation that affect either the interpretation or the existence of the measurements associated with the clinical question of interest.” [5]. We must specify both the list of potential events and the associated handling strategies. The ICH E9(R1) addendum suggests five possible strategies that may or may not be employed: treatment policy, hypothetical, composite variable, while on treatment, and principal stratum. We may apply a different handling strategy for each intercurrent event within a given estimand. In our TB estimand specification proposal, we identified a set of 35 intercurrent and missing data events that are reasonable to expect to occur in late-phase TB trials (S1 Table). Note that, although withdrawal and loss to follow-up after treatment discontinuation or treatment do not technically meet the formal definitions of *intercurrent* events, they are *missing data* events that must be handled in the statistical analysis and are therefore addressed in our approach.

(5) For the fifth attribute, population-level summary, we specify a measure of treatment effect. In the TB clinical endpoint context, we consider the difference in risk of unfavorable clinical outcomes (absence of durable cure) at a fixed time point, such as the end of follow-up, comparing participants who received an experimental regimen against standard of care.

Our TB estimand proposal recognizes the unique preferences of different stakeholders in defining a treatment effect. We defined four estimands distinguished by the application of a unique combination of handling strategies for the 35 potential intercurrent and missing data events. Table 1 provides an overview of each estimand including the intention, use in historic TB clinical trials, and appropriate statistical estimation methods and assumptions.

**Table 1:** Summary of estimands

**Estimand**	TB-specific Estimand	Composite Estimand	Assessable Estimand	Per-Protocol Estimand
**Defining characteristic**	Focuses on the effect of the treatment exclusively on TB disease outcomes.	Cautious, assuming a worst-case scenario. Lack of evidence of cure is assumed to be absence of cure.	A middle group between the TB-specific and composite estimands, providing a link to previous trials	Targets the treatment effect among the (unknown) sub-group of TB patients that take an adequate treatment course.
**Intention**	To disaggregate efficacy events from non-TB-related adverse events and other events.	To provide a cautious estimate of the treatment effect assuming many intercurrent events are indicative of absence of cure, following a strict “intention-to-treat” approach.	To provide a treatment effect estimate more closely aligned with previous trial analyses.	To provide the treatment effect in the group of participants who comply with key components of the protocol including treatment adherence.
**Potential target stakeholders**	Product developers and asset owners, research scientists, guidelines development groups, clinicians seeking to understand the efficacy of TB treatments.	National and regional TB programs, researchers seeking to compare results against previous clinical trial data, patients seeking a practical treatment effect when deciding whether to take a given TB treatment.	Researchers seeking to compare results against previous clinical trial data.	Clinicians deciding whether to prescribe a given TB treatment.
**Uses in previous TB clinical trials**	Similar to Failure or Relapse analysis in STREAM stages 1 [8] and 2 [9]	Often labeled “Strict ITT” [7] or Microbiologically Eligible [3]	Similar to a “Modified Intention-to-treat” [7, 10, 11] or “Assessable” [3] analyses.	A similar target to per-protocol analyses in previous trials, but a different estimation method more correct to the “per-protocol” label [12].
**Estimand attributes**				
1. **Treatments being tested and compared**	Trial-specific (should align with the experimental and control/standard of care regimens offered to participants through the trial)			
2. **Target population**	Trial-specific (may be shaped by the trial inclusion and exclusion criteria)			
3. **Participant-level endpoint**	Trial-specific (protocol-defined definition to determine favorable or unfavorable long-term clinical efficacy)			
4. **Specification of ICEs and associated handling strategies**	See Table S1 for complete list of ICEs and associated handling strategies. Handling strategies: • Treatment policy • Composite • Hypothetical	See Table S1 for complete list of ICEs and associated handling strategies. Handling strategies: • Treatment policy • Composite	See Table S1 for complete list of ICEs and associated handling strategies. Handling strategies: • Treatment policy • Composite • Hypothetical	See Table S1 for complete list of ICEs and associated handling strategies. Handling strategies: • Treatment policy • Composite • Hypothetical Principal Stratum
**Estimation methods with assumptions, particularly in handling of missing or censored outcomes.**	1. Multiple imputation - Assumes the ICE occurred at random given the observed data and covariates used in the model (data are “missing at random”) 2. IPCW - No unmeasured confounders associated with censoring - Censoring is not associated with outcome determination conditional on the covariates used in the model	1. No outcomes are missing or censored, estimation based on simple proportion.	1. Multiple Imputation - Assumes the ICE occurred at random given the observed data and covariates used in the model (data are “missing at random”) 2. IPCW - No unmeasured confounders associated with censoring - Censoring is not associated with outcome determination conditional on the covariates used in the model	1. Bayesian framework (for principal stratum strategy) - Applies multiple imputation for hypothetical strategy ICEs. o Assumes the ICE occurred at random (data are “missing at random”) - 3 standard assumptions for partial identifiability of estimand (joint exchangeability, monotonicity, consistency) - Specification of prior distribution reflective of model assumptions
5. **Population Summary Measure and estimation method**	Risk difference - Cochrane Mantel Haenszel o Naïve approach assuming hypothetical ICEs are durable cure o MI - Kaplan-Meier o Naïve approach censoring participants with hypothetical ICEs at the time of the ICE occurrence o MI o IPCW	Risk difference - Cochrane Mantel Haenszel - Kaplan-Meier	Risk difference - Cochrane Mantel Haenszel o Naïve approach assuming hypothetical ICEs are durable cure o MI - Kaplan-Meier o Naïve approach censoring participants with hypothetical ICEs at the time of the ICE occurrence o MI o IPCW	Risk difference - Bayesian Framework o Individual membership within principal strata is based on the unobservable distribution of ICEs given the observed and counterfactual intervention assignments. The estimand is not fully identifiable, but inference is obtained by placing Bayesian priors reflective of the necessary assumptions on the probability model.

*TB* tuberculosis, *ICE* intercurrent event, *IPCW* inverse probability of censoring weighting, *ITT* intention to treat

#### TB-specific efficacy Estimand

The TB-specific Estimand disaggregates TB-specific efficacy events from adverse or other events due to factors unrelated to TB disease. This estimand is intended to address the treatment effect for product developers who are interested in the TB-specific efficacy events for their drug or drug regimen disentangled from safety issues. We are interested in the treatment effect if everyone took their assigned regimen as specified. We apply the hypothetical strategy to many events to consider what the outcome would have been for participants had they not experienced the given (non-TB disease or treatment related) intercurrent event.

#### Composite Estimand

The Composite Estimand assumes unknown outcomes due to the occurrence of an intercurrent event are indicative of an absence of a long-term favorable outcome. It targets the programmatic question of interest and closely aligns with the legacy “intention to treat” principle. We apply only the composite and treatment policy strategies, therefore making “worst case” explicit endpoints assignments in the occurrence of an intercurrent event rather than relying on advanced statistical methods for hypothesizing what would have occurred.

#### Assessable Estimand

The Assessable Estimand is similar to the composite estimand but distinguishes missing data events relating to loss to follow-up and withdrawal after treatment completion from other types of events. These events are handled with the hypothetical strategy. This estimand aims to emulate the analyses of historic TB treatment trials [3, 7, 10, 11].

#### Per-protocol Estimand

The Per-protocol Estimand seeks to replicate the legacy “per protocol” population analysis using a causal framework rather than a simple subgroup analysis. It identifies the treatment effect in the group of participants that would have complied with the protocol and adhered to the assigned treatment, whatever that assignment may have been. We explore a mixture of handling strategies including hypothetical and principal stratum. The statistical methods for estimation of this estimand are therefore more advanced and require consideration of statistical assumptions.

### Estimation methods

The estimand defines the “what” of a treatment effect estimate; the estimation method defines the ‘how’. Estimation is informed by the strategies necessary to handle the ICEs and the method for estimating the population summary measure. The treatment policy strategy for handling intercurrent events ignores the occurrence of an event when determining the endpoint definition. The composite strategy incorporates the occurrence of the event into the endpoint definition. The hypothetical and principal stratum strategies are implemented by considering reasonable assumptions and applying one of several statistical methods.

For the composite strategy, the occurrence of the intercurrent event is mapped predominantly to the absence of durable cure or, under certain circumstances, to the presence of durable cure. For the treatment policy strategy, the occurrence of the intercurrent event is ignored when estimating the treatment effect; we use the observed participant endpoint (when available) regardless of whether or not the participant experienced this intercurrent event.

The hypothetical strategy considers what a participant’s endpoint would have been under the counterfactual, unobserved, scenario in which the intercurrent event had not occurred. Estimation methods to implement this strategy take into account the uncertainty introduced in the exercise of considering something truly unknown. Two statistical methods for implementing the hypothetical strategy are multiple imputation and inverse probability of censoring weighting (IPCW) (S1 Text). These methods are valid under both missing completely at random (MCAR) and missing at random (MAR) missing data patterns. [13] Data are MCAR if the occurrence of being observed or missing is independent of the values of the data while data are MAR if missingness is dependent on the observed values of the data. The principal stratum strategy uses the occurrence and counterfactual occurrence of intercurrent events to define the population of participants targeted by the clinical question. Within a causal framework, each participant is assigned to a “causal type” (principal stratum) with respect to the counterfactual occurrence of ICEs for each level of treatment. The population of interest can then be defined relative to the principal strata of interest; in this case, those who would not experience an ICE under either treatment assignment. One approach to effect estimation is through a Bayesian statistical model in which we set a prior distribution to incorporate model assumptions, such as monotonicity in the probability of ICE occurrence across the levels of treatment (S2 Text).

For the population summary measure of difference in risk of unfavorable clinical outcome, we can use the Cochrane Mantel Haenszel approach or the Kaplan-Meier estimator, incorporating a time component and administrative censoring at the time of the ICE occurrence.

## Illustrative example: REMoxTB trial

REMoxTB was a phase III randomized, placebo-controlled trial to assess the non-inferiority of two 4-month moxifloxacin-containing regimens against a 6-month standard control regimen. [7] The primary endpoint was the proportion of participants who experienced a composite unfavorable outcome defined by bacteriologically or clinically defined failure or relapse within 18 months after randomization. The non-inferiority margin was a between-group difference of 6 percentage points. Estimation of the treatment effect used a generalized linear model with identity-link function adjusted for stratification variables of weight group and study center. The trial presented a Bonferroni-corrected two-sided 97.5% confidence interval for treatment effect estimates. Events such as reinfection, change of treatment, and inadequate treatment determined inclusion/exclusion from the modified intention-to-treat (mITT) and per-protocol analysis populations. A total of 1931 participants were randomized in a 1:1:1 ratio to the three treatment arms (4-month isoniazid arm, 4-month ethambutol arm, and 6-month standard control arm). Non-inferiority was not shown for either experimental regimen in either of the co-primary modified intent-to-treat nor per-protocol analyses.

## Methods

We received the REMoxTB trial data from the TB-PACTS repository and reanalyzed the individual participant level data according to each of the four estimands and in the original mITT population. [TB-PACTS; https://c-path.org/programs/tb-pacts/] As a population summary measure of treatment effect, we estimated the difference in risk of unfavorable clinical outcome at 18 months after randomization. We separately compared the two experimental arms, the isoniazid arm and ethambutol arm, against the control arm using 97.5% confidence intervals and the same non-inferiority margin of 6 percentage points from the original trial analysis. As in the original trial, our analysis set excluded participants with demonstrated drug resistance at baseline, a protocol violation at the time of enrollment, and those who had no positive TB cultures within the first 2 weeks on study. Our core unfavorable outcome definition was the failure to achieve durable cure evidenced by bacteriological or clinical relapse by the end of follow-up (18 months). For each participant, we used the pre-specified list of 35 potential intercurrent and missing data events and determined whether any event had occurred during follow-up.

For each estimand, we applied statistical methods to handle events and/or to estimate the population summary measure. For the composite estimand, there is no need to apply statistical methods for handling ICEs because all ICEs are mapped to either absence or presence of durable cure. We estimated the difference in risk with the Cochrane Mantel Haenszel and Kaplan-Meier estimators.

For the TB-specific and Assessable estimands, we first applied multiple imputation and IPCW methods to handle the hypothetical strategy ICEs. For multiple imputation, we included the following baseline covariates: treatment arm, presence of chest x-ray cavities, HIV status, study center, weight band, indicator of adherence, smoking status, CD4 count, age, sex, BMI, baseline days to positivity on MGIT and demonstrated drug resistance to streptomycin, ethambutol, pyrazinamide, rifampin, moxifloxacin, or isoniazid. We generated 10 multiply-imputed complete datasets. With multiple imputation, we assume that the ICE occurred at random given the observed data and covariates used in the MI model. We computed the inverse probability of censoring weightings (IPCW) with treatment arm and weight band and applied a 1% upper and lower truncation. With IPCW we assume there are no unmeasured confounders associated with censoring and that censoring is not associated with outcome determination conditional on the covariates used in the model. When using multiple imputation, we estimated the difference in risk with the Cochrane Mantel Haenszel and Kaplan-Meier estimators. When using IPCW, we are only able to estimate the risk difference with the Kaplan-Meier estimator.

As a naïve sensitivity analysis for these two estimands, we assumed the best-case scenario (durable cure) for participants with ICEs that should be handled with the hypothetical strategy. We used the Cochrane Mantel-Haenszel method (assuming these participants experienced durable cure) and the Kaplan-Meier estimator (assuming these participants were censored at the time of the ICE and did not have an unfavorable outcome).

For the per-protocol estimand, we used a Bayesian statistical model to estimate the risk difference in the counterfactual subpopulation of participants who would not have experienced an ICE when assigned to either treatment or the standard of care. To handle ICEs with the hypothetical strategy, we again used multiple imputation (as described above) and analyzed each of the 10 imputed datasets using the Bayesian statistical model to estimate the posterior risk difference. Results were pooled across the imputed datasets to obtain a single summary estimate and confidence interval.

Finally, as a comparator, we re-analyzed the REMoxTB mITT population and estimated the difference in risk of failure to achieve durable cure with the Cochrane Mantel Haenszel and Kaplan-Meier estimators.

## Results

**Fig. 1:**
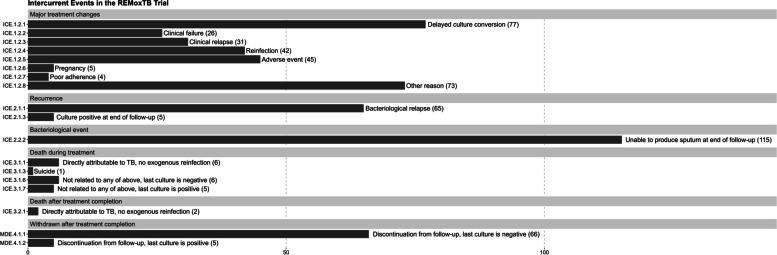
Occurrence of intercurrent and missing data events in REMoxTB trial

We analyzed individual-level data for 1785 participants who met the analysis set inclusion criteria. Among these participants, 1206 (68%) experienced durable cure and 579 (32%) experienced one of 17 ICEs from our listing (Fig. 1). The leading ICE (*n* = 115, 6%) was the inability to produce sputum at the end of the 18-month follow-up period, having sustained culture negativity at the time the last sputum culture was obtained. Other common ICEs included major treatment changes due to delayed culture conversion (*n* = 77, 4%), major treatment changes due to other reasons (*n* = 73, 4%), TB recurrence due to bacteriological relapse (*n* = 65, 4%), and withdrawal or loss to follow-up after treatment completion with last culture being negative (*n* = 66, 4%). There were limited occurrences of ICEs with handling strategies that differ across estimands. Among ICEs that are treated by at least 2 different strategies across the four estimands, “discontinuation from follow-up, last culture is negative” had the most occurrences (*n* = 66) and the incidence was similar across treatment arms (24 among control participants, 26 among isoniazid arm participants, and 16 among ethambutol arm participants).

With the composite estimand, we are able to assign an outcome to all participants based on observed data. For the TB and assessable estimands, there were 242 (14%) and 231 (13%) of participants with intercurrent/missing data events handled with the hypothetical strategy and therefore invoking analysis methods of multiple imputation and IPCW weighting. Among the variables used for multiple imputation, the proportion of complete data was: treatment arm (100%), presence of chest x-ray cavities (90%), HIV status (100%), study center (100%), weight band (100%), indicator of adherence (100%), smoking status (100%), CD4 count (7%), age (70%), sex (100%), BMI (100%), baseline days to positivity on MGIT (97%) and demonstrated drug resistance to streptomycin (98%), ethambutol (98%), pyrazinamide (98%), rifampin (99%), moxifloxacin (99%), or isoniazid (99%). For IPCW, we had complete data available for both treatment arm and weight band. For the TB-specific Estimand, the mean (standard deviation) of the truncated IPCW weights was 1.11 (0.06) for the Isoniazid versus Standard of Care comparison and was 1.09 (0.05) for the Ethambutol versus Standard of Care comparison. For the assessable estimand, the mean (standard deviation) of the truncated IPCW weights was 1.10 (0.05) for the Isoniazid versus Standard of Care comparison and was 1.08 (0.04) for the Ethambutol versus Standard of Care comparison.

**Fig. 2:**
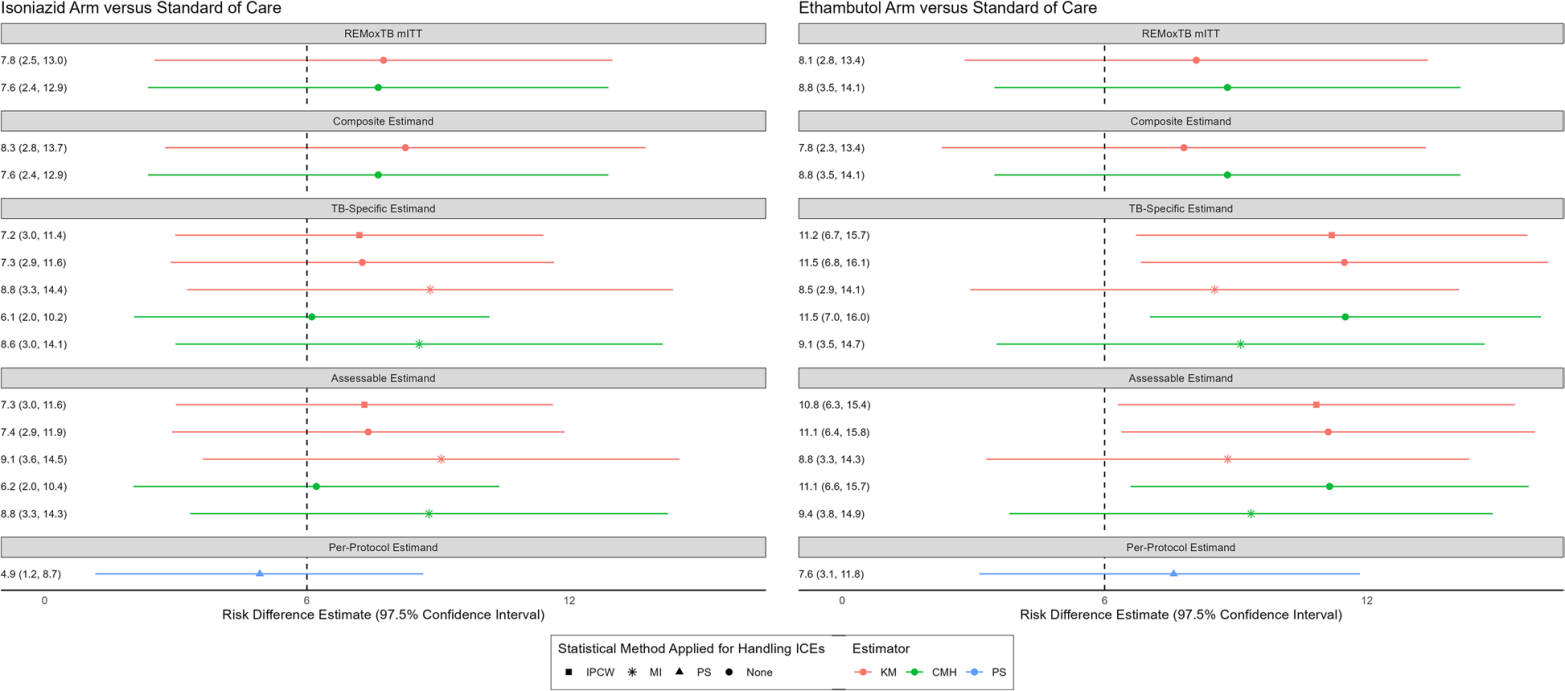
Point-range plot of risk difference estimates according to each estimand/estimation method. Each row corresponds with unique analyses within a given estimand. The vertical dotted line represents the non-inferiority margin of 6%. The results are shown as a point estimate of a risk difference and a corresponding 97.5% confidence interval. Results in orange are estimated with the Kaplan-Meier estimator (KM), results in green are estimated with the Cochrane Mantel Haenszel method (CMH), and results in blue are estimated according to the Principal Stratum method (PS). Point estimates represented by a square have implemented Inverse Probability of Censoring Weighting (IPCW) to handle certain intercurrent events. Point estimates represented by an asterisk have implemented Multiple Imputation (MI) to handle certain intercurrent events. Points estimates represented by a triangle have implemented the Principal Stratum (PS) method to handle certain intercurrent events. The remaining point estimates are represented by a circle meaning that no special statistical methods were used to handle intercurrent events

We consistently found an absence of non-inferiority for both the isoniazid and ethambutol regimens compared with standard of care for all estimands and methods of estimation (Fig. 2). These findings are consistent with the published ReMoxTB trial analysis. The point estimates of the treatment effect measures were similar across all estimands and methods of estimation. For all estimands and methods of estimation, the risk difference was larger for the ethambutol arm versus standard of care as compared with the risk difference for the isoniazid arm versus standard of care (as was also shown in the primary REMoxTB analyses). [7] Using multiple imputation resulted in larger variance estimates (wider confidence intervals) than inverse probability of censoring weighting or naïve censoring.

## Discussion

We have demonstrated an application of our proposed estimands for the primary efficacy objective in TB treatment trials using the REMoxTB randomized trial as a case study and have described appropriate methods for estimation. Our estimands gave consistent conclusions in agreement with the published trial findings. Applying more complex statistical analysis methods did not lead to sizable differences in the estimates of the population summary measure of treatment effect. With our findings in mind, we anticipate that future TB treatment trials could consider using one (or two) of our proposed estimands as primary and perhaps include others as secondary. The choice of estimands will depend on the overall objective and target audience specific to a given trial and it will also be driven by the assumptions and complexities required for estimation.

Our re-analyses of REMoxTB with our 4 estimands lead to consistent conclusions aligned with the published trial findings and the reanalysis in the mITT population. This gives further confirmation of the REMoxTB trial results. The variability in estimates of the population summary measure of treatment effect is driven by the different statistical assumptions, methods implemented, and numbers of participants experiencing ICEs. It is important to understand that these estimands answer slightly different questions and that no single estimand gives a more true or less biased treatment effect estimate; our objective was to identify appropriate methods of estimation for each estimand as well as compare deviations between estimands.

Our application using this historic trial data has limitations. Only about half of the anticipated intercurrent events from our proposal actually occurred in the REMoxTB trial. We cannot say whether this will be typical in future trials. Furthermore, in REMoxTB, there were limited occurrences of intercurrent events that are handled with different strategies across the four proposed estimands. If these intercurrent events are more frequent in other settings, then the different estimands or estimation methods may result in greater variability of the point estimates and confidence intervals. When retrofitting the estimands, we did not have all essential data available to make determinations about the occurrence of some intercurrent events. In many cases, we were able to determine that an intercurrent event had occurred but relevant outcome information was not available beyond the occurrence. We assumed that the intercurrent event occurred at the time the original trial determined the favorable/unfavorable outcome. Future trials using our estimands should ensure that case report forms collect all of the necessary information to make outcome determinations and collect clinically relevant information during the course of follow-up for statistical models such as the multiple imputation model. When applying this framework in a future trial, it is possible that a participant will experience multiple ICEs including a situation where the first ICE is an event handled with the treatment policy strategy followed by another ICE handled with one of the other handling strategies.

Our specification of estimands (v1.0) proposed for the application of ICH E9 (R1) concepts in the TB treatment trial context is a first attempt at defining estimands for this use and is an evolving piece of work where we have now initiated the conversation and demonstrated its use. [6] We have revised the proposal in parallel with the work for this analysis and anticipate that, as future trials use our estimands, new challenges or ideas may arise and possibly lead to additional revisions or considerations. Others have recently considered the use of well-specified estimands for TB trials, offering different perspectives. [14, 15] None of our estimands uses the “while on treatment” (or “while on study”) handling strategy as we did not have need of this strategy in the TB treatment trial context where long-term post-treatment follow-up is of the most importance when determining the outcome of interest. We will continue to update the estimand proposal in light of these and other results, and welcome further input and collaborators in the spirit of open research (https://osf.io/4a7cq/).

Finally, it is beyond the scope of this paper to address recommendations for preferred estimands or statistical estimation methods based on objective numeric evidence. However, our reanalysis according to each estimand and estimation method revealed that implementation of some estimands was less complex and required fewer statistical assumptions while yielding similar results. The composite estimand is simple to implement and requires few estimation assumptions but produces a cautious estimate of the treatment effect that may not fairly answer the trial objective. The TB-Specific and Assessable estimands require assumptions about missing data and use statistical methods to impute participant outcomes under the hypothetical counterfactual scenario in which an ICE did not actually occur. However, these estimands more adequately disaggregate true TB efficacy events from non-TB-related AEs. The per-protocol estimand is especially complex to estimate and requires high-level statistical assumptions. However, this estimand should be admired for assessing a true per-protocol effect within a causal framework, in contrast with legacy per-protocol analyses that are essentially simple lop-sided subgroup analyses. Across all estimands, the advanced statistical methods required slightly more thought and computational time but should not be a barrier to implementation. The statistical methods are available in common software including R, SAS, and Stata. While we did not find meaningful advantages to implementing more complex statistical estimation methods, future trials with higher proportions of certain intercurrent events may see apparent differences in results. In future work, we will address this by comparing the estimands and methods of estimation in a broad simulation study under an array of different settings.

## Conclusions

Our proposed estimand framework aligns with ICH E9(R1) and gives trialists a thorough starting point for estimand specification when designing future TB treatment randomized controlled trials. We have demonstrated its use and discussed methods for estimation. This exercise may be useful to complete in other recent TB treatment trials as additional sensitivity analyses confirming trial results and to continue refining the proposed estimands and estimation methods. We recommend that future trials utilize this framework in an effort to reduce variability in trial outcome definitions and thereby facilitate more insightful between trial comparisons.

### Supplementary Information

**Additional file 1: S1 Table. **Strategies for handling intercurrent and missing data events for each estimand (Table 1 from Section 6 of the estimand proposal).

**Additional file 2: S1 Text. **Methods for Multiple Imputation and Inverse Probability of Censoring Weighting.

**Additional file 3: S2 Text. **Methods and technical details for principal stratum estimand and estimation.

## Data Availability

The estimand specification material is available publicly (10.17605/OSF.IO/4A7CQ). The datasets analyzed during the current study are available in the TB-PACTS repository (https://c-path.org/programs/tb-pacts/).
