# Supplementary material for: Estimands for clinical endpoints in tuberculosis treatment randomized controlled trials: a retrospective application in a completed trial
Source: Trials. 2024 Mar 12;25:180. doi: 10.1186/s13063-024-07999-w (PMC10929173; doi:10.1186/s13063-024-07999-w)
Supplement: Supplementary file 1 — Additional file 1: S1 Table. Strategies for handling intercurrent and missing data events for each estimand (Table 1 from Section 6 of the estimand proposal). [file 13063_2024_7999_MOESM1_ESM.docx]

S1 Table. Strategies for dealing with intercurrent events and missing data events for each estimand

|  | | *TB-specific  Estimand* | *Composite  Estimand* | *Assessable  Estimand* | *Per-Protocol  Estimand* |
| --- | --- | --- | --- | --- | --- |
| **1 TREATMENT EVENTS** | |  |  |  |  |
| **1.1 Minor treatment changes** | |  |  |  |  |
| ICE.1.1.1 | Dose modifications | Treatment policy | Treatment policy | Treatment policy | Treatment policy |
| ICE.1.1.2 | Frequency modifications | Treatment policy | Treatment policy | Treatment policy | Treatment policy |
| ICE.1.1.3 | Stop a drug without starting a new one | Treatment policy | Treatment policy | Treatment policy | Treatment policy |
| ICE.1.1.4 | Extension of treatment to make up for missed doses | Treatment policy | Treatment policy | Treatment policy | Treatment policy |
| ICE.1.1.5 | Temporary drug re-challenge fewer than 14 days | Treatment policy | Treatment policy | Treatment policy | Treatment policy |
| **1.2 Major treatment changes** | |  |  |  |  |
| *Addition of at least one off-study drug because of…* | | |  |  |  |
| ICE.1.2.1 | Delayed culture conversion | Composite | Composite | Composite | Composite |
| ICE.1.2.2 | Clinical failure | Composite | Composite | Composite | Composite |
| ICE.1.2.3 | Clinical relapse | Composite | Composite | Composite | Composite |
| ICE.1.2.4 | Reinfection | Hypothetical | Composite | Hypothetical | Hypothetical |
| ICE.1.2.5 | Adverse event | Hypothetical | Composite | Hypothetical | Hypothetical |
| ICE.1.2.6 | Pregnancy | Hypothetical | Composite | Hypothetical | Hypothetical |
| ICE.1.2.7 | Poor adherence | Hypothetical | Composite | Composite | Principal stratum |
| ICE.1.2.8 | Other reason | Hypothetical | Composite | Hypothetical | Principal Stratum |
| **1.3 Discontinuation from treatment** | |  |  |  |  |
| ICE.1.3.1 | Fail to complete adequate treatment without changes or restart | Treatment policy | Treatment policy | Treatment policy | Principal stratum |
| **2 BACTERIOLOGICAL EVENTS** | |  |  |  |  |
| **2.1 Recurrence** | |  |  |  |  |
| ICE.2.1.1 | Bacteriological relapse | Composite | Composite | Composite | Composite |
| ICE.2.1.2 | Bacteriological recurrence in the absence of strain typing | Composite | Composite | Composite | Composite |
| ICE.2.1.3 | Culture positive at end of follow-up | Composite | Composite | Composite | Composite |
| ICE.2.1.4 | Reinfection without change in treatment | Treatment Policy | Treatment Policy | Treatment Policy | Treatment Policy |
| **2.2 Other bacteriological events** | |  |  |  |  |
| ICE.2.2.1 | Isolated positive culture | Treatment policy | Treatment policy | Treatment policy | Treatment policy |
| ICE.2.2.2 | Unable to produce sputum at end of follow-up | Composite (cure) | Composite (cure) | Composite (cure) | Composite (cure) |
| **3 DEATH** | | | | | |
| **3.1 Death During treatment** | |  |  |  |  |
| ICE.3.1.1 | Directly attributable to TB, no exogenous reinfection | Composite | Composite | Composite | Composite |
| ICE.3.1.2 | Directly attributable to TB, exogenous reinfection | Hypothetical | Composite | Hypothetical | Hypothetical |
| ICE.3.1.3 | Suicide | Hypothetical | Composite | Composite | Hypothetical |
| ICE.3.1.4 | Accident or trauma (not suicide) | Hypothetical | Composite | Hypothetical | Hypothetical |
| ICE.3.1.5 | Considered related to study treatment | Hypothetical | Composite | Composite | Hypothetical |
| ICE.3.1.6 | Not related to any of above, last culture is negative | Hypothetical | Composite | Composite | Hypothetical |
| ICE.3.1.7 | Not related to any of above, last culture is positive | Composite | Composite | Composite | Composite |
| **3.2 Death After treatment completion** | | | | | |
| ICE.3.2.1 | Directly attributable to TB, no exogenous reinfection | Composite | Composite | Composite | Composite |
| ICE.3.2.2 | Directly attributable to TB, exogenous reinfection | Hypothetical | Composite | Hypothetical | Hypothetical |
| ICE.3.2.3 | Suicide | Hypothetical | Composite | Hypothetical | Hypothetical |
| ICE.3.2.4 | Accident or trauma (not suicide) | Hypothetical | Composite | Hypothetical | Hypothetical |
| ICE.3.2.5 | Not related to any of above, last culture is negative | Hypothetical | Composite | Hypothetical | Hypothetical |
| ICE.3.2.6 | Not related to any of above, last culture is positive | Composite | Composite | Composite | Composite |
| **4 WITHDRAWAL OR LOSS TO FOLLOW-UP AFTER TREATMENT DISCONTINUATION OR COMPLETION  (Missing Data Events)** | | | | | |
| MDE.4.1.1 | Discontinuation from follow-up, last culture is negative | Hypothetical | Composite | Hypothetical | Hypothetical |
| MDE.4.1.2 | Discontinuation from follow-up, last culture is positive | Composite | Composite | Composite | Composite |
