# Supplementary material for: Estimands for clinical endpoints in tuberculosis treatment randomized controlled trials: a retrospective application in a completed trial
Source: Trials. 2024 Mar 12;25:180. doi: 10.1186/s13063-024-07999-w (PMC10929173; doi:10.1186/s13063-024-07999-w)
Supplement: Supplementary file 2 — Additional file 2: S1 Text. Methods for Multiple Imputation and Inverse Probability of Censoring Weighting. [file 13063_2024_7999_MOESM2_ESM.docx]

# S1 Text: Hypothetical Strategy

To apply the hypothetical strategy, we use statistical methods to assign what a participant’s endpoint determination would have been under the counterfactual, unobserved, scenario in which the intercurrent event had not occurred, taking into account the uncertainty in this process of assignment. Two available statistical methods are multiple imputation and inverse probability of censoring weighting.

Multiple imputation is a procedure readily available with standard computing packages, such as the MICE package in R. [10] For participants who experience one of the intercurrent events marked for hypothetical handling, we impute the binary outcome value using other observed participant characteristics associated with durable cure. We assume that the intercurrent event occurred at random (analogous to missing-at-random pattern of missing data). We generate M multiply imputed, complete datasets in which those who experienced an intercurrent event now have imputed outcome values. Using each of the M complete datasets, we estimate the population-level summary measures and apply Rubin’s Rule to obtain the pooled estimate and variance over all M datasets. One limitation of this approach is that we impute only the binary outcome and not the time-to-event component. Thus, we assume the time of the intercurrent event occurrence is the same as the time of the determined presence/absence of durable cure endpoint.

Inverse probability of censoring weighting (IPCW) is also available with standard computing packages, such as the IPW package in R. [11] We calculate participant-level weights as the inverse probability of the occurrence of an intercurrent event then apply the weights in a weighted Cox proportional hazards model. This method gives more weight to participants with a fully observed endpoint who share similar profiles as those participants who experienced the intercurrent event and therefore had an unobserved endpoint.
