# Supplementary material for: Estimands for clinical endpoints in tuberculosis treatment randomized controlled trials: a retrospective application in a completed trial
Source: Trials. 2024 Mar 12;25:180. doi: 10.1186/s13063-024-07999-w (PMC10929173; doi:10.1186/s13063-024-07999-w)
Supplement: Supplementary file 3 — Additional file 3: S2 Text. Methods and technical details for principal stratum estimand and estimation. [file 13063_2024_7999_MOESM3_ESM.docx]

# S2 Text: Principal Stratum Strategy

The principal stratum strategy (also referred to as principal stratification) takes a different approach to handling ICEs from any of the other strategies described. Instead of considering the role the ICE may play with respect to the outcome (and whether it should be imputed or counterfactually handled), it uses ICEs to define the population of patients targeted by the clinical question. Within a causal framework, each individual is assigned to a ‘causal type’ (referred to as a principal stratum) that is defined with respect to the (counterfactual) occurrence of ICEs for each level of treatment that could be (counterfactually) assigned. For example, an individual who would never experience an ICE on any of the treatment levels would belong to the “immune” principal stratum. Similarly, an individual who experiences an ICE on all levels of treatment would belong to the “doomed” principal stratum. The target of estimation is then defined with respect to principal stratum membership. As an example, the target estimand may be the intervention effect among those who are “immune” to ICEs, e.g., those who would not experience an ICE under any intervention assignment. Principal stratum estimands are not restricted to one of the principal strata and can accommodate a wide-range of scientifically relevant questions of interest.

One method of estimation when implementing the principal stratum strategy is through a Bayesian statistical model. With this model, there are 3 implicit assumptions: (1) joint exchangeability, (2) consistency, and (3) monotonicity. Joint exchangeability is reasonable to assume in the context of a randomized trial because it assumes treatment assignment is jointly independent of the potential outcomes. Consistency is a standard assumption in casual inference methods. It is reasonable to assume that an individual’s potential outcome is equal to their observed outcome when their counterfactual history is equivalent to their observed history. The monotonicity assumption can be partially assessed statistically by comparing the proportion of ICEs among the treatment arms being compared.

Monotonicity, in this setting, eliminates the “harmed” principal stratum for the sake of identifiability. It implies that the proportion of ICEs occurring among those receiving the novel regimen should be no greater than the proportion of ICEs among those receiving the standard of care. This assumption was tested using the REMoxTB data, where the proportions of participants experiencing an ICE on the HRZE, 2MHRZ/2MHR, and 2EMRZ/2MR were 0.863, 0.841, and 0.891, respectively. Hence, the assumption was not rejected for the comparison with 2MHRZ/2MHR but did not hold for 2EMRZ/2MR. As a result, the Bayesian model was run with three distinct priors on the “harmed” strata enforcing 1) strong monotonicity, 2) weak monotonicity, and 3) no monotonicity. Results under the “weak” and “no” monotonicity assumptions were more conservative, but did not change meaningfully from the results presented in this manuscript which assumed “strong” monotonicity (Fig. S1).

| 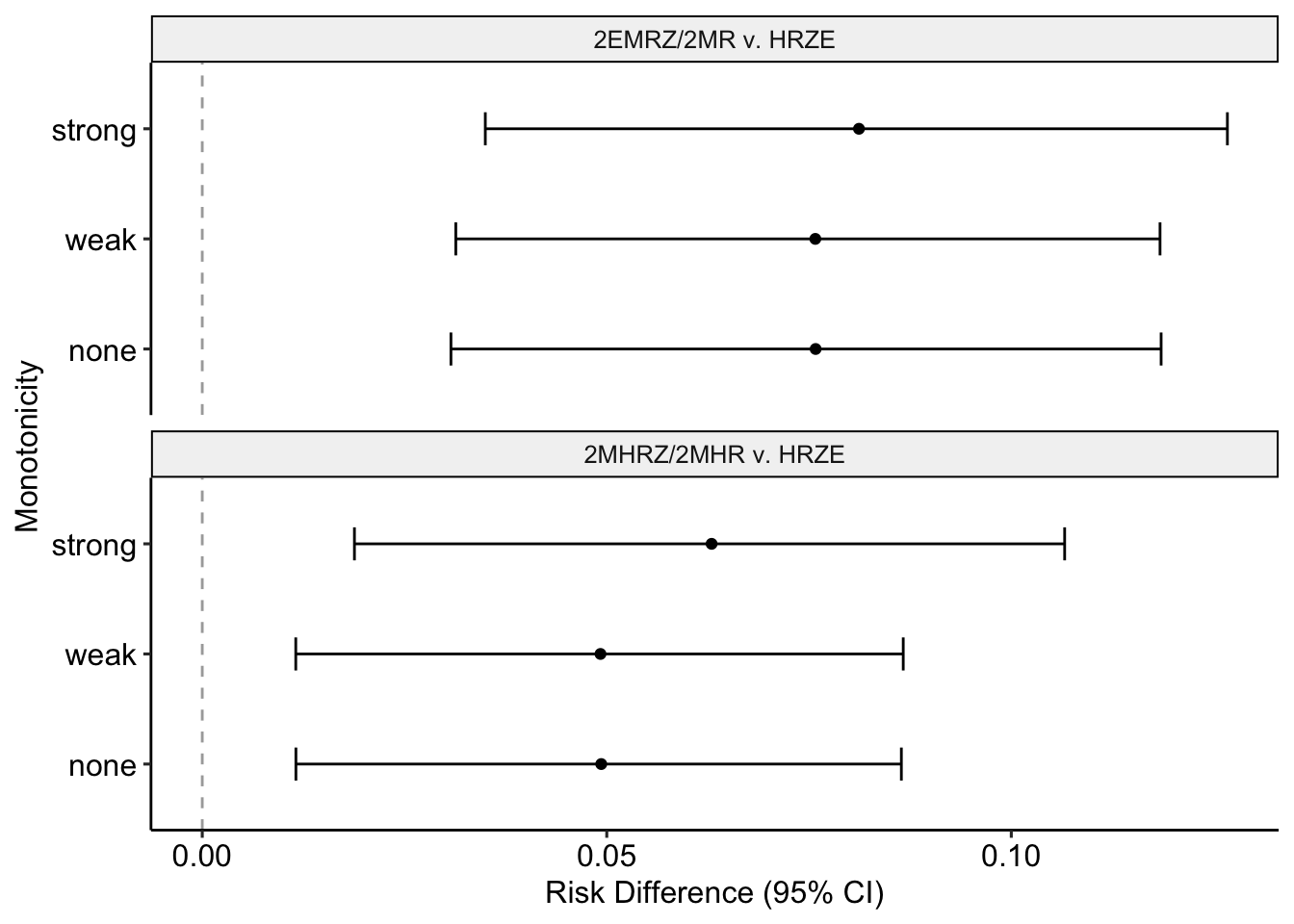 |
| --- |
| **Figure S1.** Risk difference (95% CI) estimates under the different monotonicity assumptions for the comparisons of 2EMRZ/2MR versus HRZE and 2MHRZ/2MHR versus HRZE. |

The flexibility both in terms of what causal questions can be asked as well as the appropriate statistical methodology and assumptions needed for identification of the target estimand are a topic of discussion in the causal inference community. [12, 13] Bayesian methods are arguably well-suited to explicitly account for the uncertainties that must be accounted for in estimating principal stratum estimands [14], though frequentist approaches have been used as well [15].
